# Selective Detection and Phylogenetic Diversity of *Acaryochloris* spp. That Exist in Association with Didemnid Ascidians and Sponge

**DOI:** 10.1264/jsme2.ME11295

**Published:** 2012-02-22

**Authors:** Satoshi Ohkubo, Hideaki Miyashita

**Affiliations:** 1 Graduate School of Human and Environmental Studies, Kyoto University, Yoshida Nihonmatsu-cho, Sakyo-ku, Kyoto 606–8501, Japan

**Keywords:** chlorophyll *d*, *Acaryochloris marina*, cyanobacteria, ascidians, DGGE

## Abstract

*Acaryochloris* spp. are unique cyanobacteria which contain chlorophyll *d* as the predominant pigment. The phylogenetic diversity of *Acaryochloris* spp. associated with 7 *Prochloron*- or *Synechocystis*-containing didemnid ascidians and 1 *Synechococcus*-containing sponge obtained from the coast of the Republic of Palau was analyzed; we established a PCR primer set designed to selectively amplify the partial 16S rRNA gene of *Acaryochloris* spp. even in DNA samples containing a large amount of other cyanobacterial and algal DNAs. Polymerase chain reaction-denaturing gradient gel electrophoresis with this primer set enabled detection of the phyogenetic diversity of *Acaryochloris* spp. All the ascidian and sponge samples contained *Acaryochloris* spp. Fourteen phylotypes that were highly homologous (98–100%) with *A. marina* MBIC11017 were detected, while only 2 phylotypes were detected with our previously developed method for detecting cyanobacteria. The results also revealed that many uncultured phylotypes of *Acaryochloris* spp. were associated with those didemnid ascidians, since a clonal culture of only 1 phylotype has been established thus far. No specific relationship was found among the *Acaryochloris* phylotypes and the genera of the ascidians even when sample localities were identical; therefore, these invertebrates may provide a favorable habitat for *Acaryochloris* spp. rather than hosts showing any specific symbiotic relationships.

Organisms belonging to the genus *Acaryochloris* are unicellular cyanobacteria, which are unique in the sense that they contain chlorophyll (Chl) *d* as the predominant pigment (14–16). Moreover, these organisms are unusual photoautotrophs that use Chl *d* as the reaction center pigments for both photosystems I and II (7, 27) and are capable of using near infrared light (700–750 nm), in addition to photosynthetically active radiation (PAR, 400–700 nm), as their sole light source (13).

The first clonal strain *Acaryochloris marina* MBIC11017 was isolated from microalgal symbionts obtained by squeezing the didemnid ascidian *Lissoclinum patella*, which contains *Prochloron* sp. as the predominant symbiont (14). Several clonal cultures of *Acaryochloris* strains were established from the cavities of *Prochloron*-harboring didemnid ascidians (Miyashita *et al.*, unpublished). Moreover, cyanobacteria containing Chl *d* were detected around didemnid ascidians (9). On the basis of these findings, it was presumed that *Acaryochloris* spp. might be widely distributed in tropical and subtropical environments where these organisms might share a symbiotic relationship with didemnid ascidians; however, *Acaryochloris* spp. have been found along the coast of Awaji Island, Japan, where they were isolated as epiphytes on marine red macroalgae (19); and also along the coast of Salton Sea, CA, USA, where they were found in a microbial mat community (12). These findings suggest that *Acaryochloris* spp. are also widely distributed as epiphytic and epilithic cyanobacteria, and raise a question regarding the existence of a specific relationship between *Acaryochloris* spp. and didemnid ascidians.

However, little is known about the genetic diversity of *Acaryochloris* spp. in natural environments. Three clonal strains of the *Acaryochloris* spp., assigned as MBIC11017, Awaji-1, and CCMEE5410, resemble one another in terms of morphology, chlorophyll composition, and 16S rRNA gene sequence (>99% similarity); however, strains MBIC11017 and Awaji-1 differ in terms of their fluorescence properties (1). Moreover, in the MBIC11017 strain, the amount of phycobiliproteins was low but certainly existed under high light conditions; however, the CCMEE5410 strain consistently lacked phycobiliproteins (3). Because of scant knowledge about the diversity and ecology of *Acaryochloris* spp., the relationship between *Acaryochloris* spp. and didemnid ascidians is unclear. The question is whether didemnid ascidians are simple habitats of *Acaryochloris* spp. without any specific symbiotic relationships, or whether *Acaryochloris* spp. have specific relationships with didemnid ascidians. In order to answer these questions, it is necessary to investigate the distribution and genetic diversity of *Acaryochloris* spp. in didemnid ascidians. This study will provide new knowledge about the evolution of cyanobacteria-ascidian symbiosis.

Polymerase chain reaction-denaturing gradient gel electrophoresis (PCR-DGGE) is one of the methods that can be used to detect the presence of microorganisms in environmental samples and investigate their genetic diversity in the environment (20). We previously developed a method for identifying epiphytic *Acaryochloris* spp. from macroalgae samples, which involved PCR-DGGE using the 2 primer sets BAC341F-CYA781R(a) and BAC341F-CYA781R(b), individually (22). In this method, DNA extracted from host-algal plastids and heterocyst-forming cyanobacteria was amplified with BAC341F-CYA781R(a), and DNA extracted from other cyanobacteria (including *Acaryochloris* spp.) was amplified with another primer set, BAC341F-CYA781R(b). With the help of this method, 2 phylotypes of *Acaryochloris* spp. that were present on red, green and brown macroalgae were detected (22).

However, didemnid ascidians known to shelter *Acaryochloris* spp. predominantly harbor organisms of *Prochloron* spp. or *Synechocystis trididemnii*, which is the closest relative of *Prochloron*, as their symbionts (9, 16, 18). Since the 16S rRNA genes of those predominant symbiotic cyanobacteria were amplified with the primer set BAC341F-CYA781R(b), the DGGE bands of *Acaryochloris* spp. may have been masked in our previously developed method; therefore, we needed a method for the selective detection of *Acaryochloris* spp. even in samples containing other predominant cyanobacteria.

In this study, we developed a primer set specific to organisms belonging to the *Acaryochloris* genus. By performing PCR-DGGE with this primer set, we were able to selectively amplify the 16S rRNA genes of *Acaryochloris* spp., and to investigate the genetic diversity of these organisms that are associated with didemnid ascidians and a sponge, which was found in the Republic of Palau. The results revealed that many phylotypes of *Acaryochloris* spp. are widely distributed and do not exhibit any host specificity.

## Materials and Methods

### Invertebrate samples

**Fig. 1:**
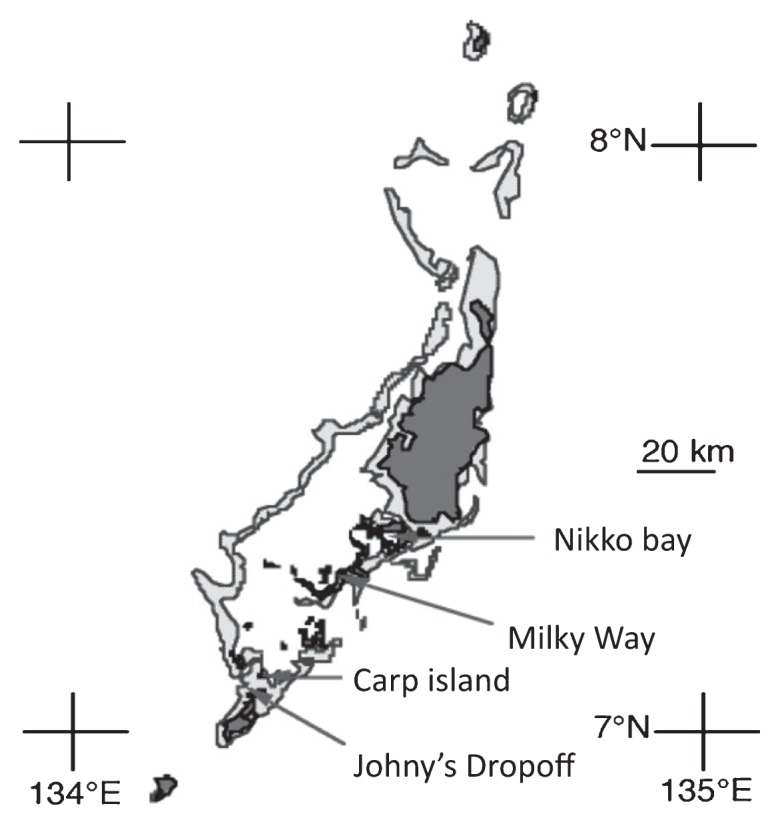
Sampling locations of didemnid ascidians and sponge.

Samples of ascidians and sponges harboring cyanobacterial symbionts were collected from the coast of the Republic of Palau in March 2004 (Fig. 1). The host species were identified in the basis of their morphological characteristics (Table 1). Each invertebrate was separately preserved in ethanol and stored at 4°C until use.

### DNA extraction

The invertebrate samples were rinsed several times with ethanol, and subsequently dried under reduced pressure. Each sample was ground to a fine powder in liquid nitrogen with a mortar and pestle. Approximately 100 mg of the ground sample was transferred into a 1.5-mL microtube containing 500 μL cell lysis buffer (100 mM Tris-HCl [pH 8.0], 50 mM EDTA, 500 mM NaCl, and 1% cetyltrimethylammonium bromide [CTAB]) and 50 μL of 10% sodium dodecyl sulfate (SDS). After incubating the microtube at 80°C for 5 min, 300 μL of 10 M potassium acetate and 6 μL proteinase K (4 mg mL^−1^ stock solution) were added, the microtube was incubated at 50°C for 60 min, put on ice for 20 min, and centrifuged at 4°C for 15 min at 16,000×*g*. The upper aqueous phase was transferred into a new microtube, and the total DNA was then extracted using a general phenol-chloroform extraction method and 2-propanol precipitation. The resultant DNA pellets were washed with 70% ethanol, air dried, and resuspended in 50 μL TE buffer (10 mM Tris-HCl [pH 8.0] and 1 mM EDTA).

### Primer design and PCR

We designed an oligonucleotide primer so as to selectively amplify the 16S rRNA genes of *Acaryochloris* spp. This primer, namely, AcaryoSPF (corresponding to positions 78–98 of the 16S rRNA gene in *E. coli*, 5′-AGGAGCTTGCTCCTTGGTGG-3′), anneals to the V1 region of the 16S rRNA gene of *A. marina*. Theoretically, by using a cyanobacteria-specific primer CYA781R(b) (corresponding to positions 781–805 of the 16S rRNA gene in *E. coli*, 5′-GACTACAGGGGTATCTAATCCCTTT-3′) along with AcaryoSPF, approximately 700-bp fragments of the 16S rRNA genes from *Acaryochloris* spp. can be amplified.

Each PCR was performed in a 20-μl reaction mixture containing approximately 50 ng of the extracted DNA, 4 pmol of each primer, 0.4 U ExTaq polymerase (Takara, Ohtsu, Japan), 1.6 μL dNTP mixture (2.5 mM each, Takara), 4 μL 5× Ampdirect-D (Shimadzu Biotech, Kyoto, Japan), and 4 μL 5× AmpAddition-3 (Shimadzu Biotech). To reduce the formation of spurious by-products during the PCR process, touchdown PCR was employed (20). After preincubation at 95°C for 5 min, 30 incubation cycles were performed. Each cycle comprised incubation at 94°C for 1 min, 65°C–55°C for 1 min (a decrease of 1°C for every alternate cycle for the first 20 cycles), and 72°C for 1 min. In this study, we used 4 different primer sets: AcaryoSPF-CYA781R(b), -BAC341F (corresponding to positions 341–357 in *E. coli*; GC clamp, 5′-CCTACGGGAGGCAGCAG-3′) -CYA781R(a) (corresponding to positions 781–805 in *E. coli*, 5′-GACTACTGGGGTATCTAATC CCATT-3′), BAC341F-CYA781R(b), and BAC341F-CYA781R (CYA781R(a) and CYA781R(b) in equimolar concentrations) (21).

After the first PCR with the primer set AcaryoSPF-CYA781R(b), the second nested PCRs were performed. After the electrophoresis of PCR products on 1.5% agarose gels in 1× TAE buffer (40 mM Tris, 20 mM acetate, and 1 mM EDTA), the gels were stained with ethidium bromide. Bands were excised from the gels with a sterile scalpel and transferred into 1.5-mL microtubes containing phenol. The DNA was eluted by subjecting it to a freeze-thaw cycle, extracted with phenol, and concentrated by ethanol precipitation. The purified DNA fragments thus obtained were used as templates, and a second round of nested PCR was performed as described above with the primer set BAC341F-CYA781R(b). By performing this nested PCR, we obtained fragments containing GC clamps, which were of suitable length for use in DGGE (~500 bp).

### DGGE

DGGE was performed by using the DCode Universal Mutation Detection System (BioRad, Hercules, CA, USA). PCR products (approximately 200 ng) were loaded onto 7% (w/v) polyacrylamide gels immersed in 0.5× TAE buffer (20 mM Tris, 10 mM acetate, and 0.5 mM EDTA). Polyacrylamide gels (7%; w/v) were prepared with denaturing gradients in the range 20–50% (100% denaturant was achieved by using 7 M urea and 40% formamide). Electrophoresis was carried out at 60°C for 12 h at 100 V (under constant voltage). After electrophoresis, the gels were stained with ethidium bromide, rinsed with water, and photographed on an ultraviolet (UV) transillumination table with a charged coupled device (CCD) camera.

### Sequencing of DGGE fragments

The DGGE bands were carefully excised from the gels with a sterile scalpel and were used for reamplification by PCR. Each band was placed individually in 50 μL TE buffer and incubated for 24 h at 4°C in order to allow the DNA fragments to diffuse into the buffer. The resultant solution was then used as a template for reamplification as described above. The reamplified PCR products were used as templates for sequencing reactions that were performed using the ABI PRISM BigDye Terminator v3.1 Cycle Sequencing Ready Reaction Kit (Applied Biosystems, Foster City, CA, USA). The products obtained were subsequently analyzed on an ABI PRISM 3100 Genetic Analyzer (Applied Biosystems), according to the manufacturer’s instructions. The sequences obtained were compared with GenBank sequences using the NCBI BLAST search tool (2), and deposited in the DDBJ database under accession numbers AB479412 to AB479463.

### Phylogenetic analysis

Cyanobacterial and chloroplast 16S rRNA gene sequences available in GenBank and those detected in this study were aligned and analyzed using the ARB software package (11). A phylogenetic tree was constructed on the basis of almost complete sequences (corresponding to positions 74–1290 in the *E. coli* genome). The positions at which the alignment resulted in gaps or ambiguities were omitted from the analysis. The neighbor-joining method applied is based on a matrix of evolutionary distances determined by the Jukes-Cantor equation and subjected to bootstrap analysis (1,000 replicates). Partial sequences containing DGGE bands were integrated into the tree according to the maximum parsimony criterion such that the topology of the tree remained unchanged.

The sequences of the *Acaryochloris* phylotypes obtained in this study and those already reported were separately aligned and analyzed with the PHYLIP package (version 3.68) (8). Bootstrap values were obtained through the SEQBOOT program (500 data sets were generated), and distance matrices were generated with the DNADIST program (Jukes-Canter matrix). Neighbor-joining analysis, maximum likelihood analysis, and maximum parsimony analysis were carried out with the NEIGHBOR, DNAML, and DNAPARS programs, respectively.

## Results

### Detection of *Acaryochloris* spp. by cyanobacteria-specific PCR-DGGE

**Fig. 2:**
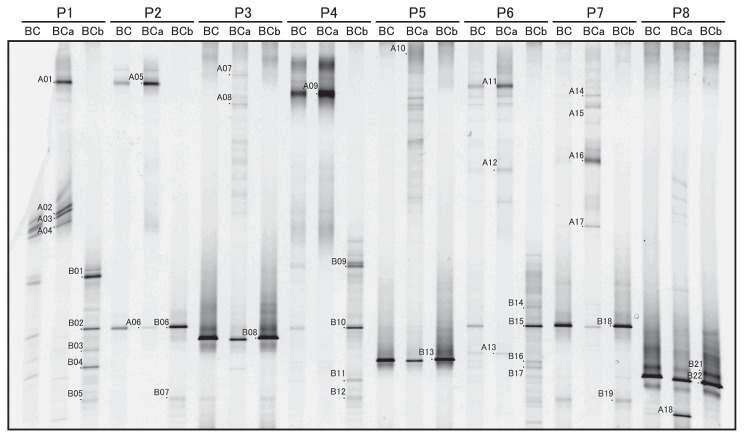
Diversity of cyanobacteria and microalgae that exist in association with didemnid ascidians and sponge. Template DNAs were extracted from 7 didemnid ascidians (P1–7) and 1 sponge (P8) (Table 1). PCR amplification was performed with 3 primer sets: BAC341F-CYA781R (BC lanes), BAC341F-CYA781R(a) (BCa lanes), and BAC341F-CYA781R(b) (BCb lanes).

PCR-DGGE analysis of the total DNA obtained from 7 ascidians and 1 sponge (Table 1) was performed with our previously developed method using 3 primer sets, namely, BAC341F-CYA781R, BAC341F-CYA781R(a), and BAC341F-CYA781R(b) (BC, BCa, and BCb lanes, respectively, in Fig. 2). All the bands detected in BC lanes were also detected in BCa or BCb lanes; therefore, we excised and sequenced bands in BCa and BCb lanes, which were numbered as indicated on the DGGE gel (bands A01–18 and B01–21 in Fig. 2). The sequences of the closest relatives that were found in the GenBank database by performing a BLAST search are listed in Table 2.

DGGE bands of *Acaryochloris* spp. were only obtained from samples of *L. patella* (P1) and *Didemnum molle* (P4) (bands B03 and B10, respectively, in Fig. 2). The sequence of band B03 was identical to that of *A. marina* MBIC11017, while the sequence of band B10 showed relatively low homology with that of *A. marina* (96.8%). In most samples, the major bands that appeared in the BC lanes were also the major bands in the BCb lanes (Fig. 2). The dominant bands were derived from the following samples of the predominant cyanobacterial symbionts of didemnid ascidians and the sponge: *Prochloron* spp. (bands B02, B08, B15, B18) in *L. patella* (P2, P6), *Lissoclinum punctatum* (P3), and *Lissoclinum timorence* (P7); *Synechocystis trididemni* (B13) in *Didemnum* sp. (P5); and *candidatus* Synechococcus spongiarum P129SC1 (B20, 21) in *Neopetrosia exigua* (P8) (Table 2). A band of *Prochloron* sp. was also obtained from the P1 sample (band B02, Fig. 2) but not from the P4 sample. *Synechococcus* spp. (bands B05, B07, B12, B19), *Xenococcus* sp. (B04), and filamentous cyanobacteria (B01, B09, B14, B16, B17) were also detected in BCb lanes. In BCa lanes, bands of 2 cyanobacteria, 9 diatom chloroplasts, and 7 unclassified bacteria were detected. The results of this experiment as well as those of a previous isolation experiment (Miyashita *et al.*, unpublished) indicate that *Acaryochloris* spp. may not be detected because their bands on the gel may have been obscured by the presence of predominant cyanobacterial symbionts.

### Construction of a specific primer for the detection of *Acaryochloris* spp

Both strains MBIC11017 and CCMEE5410 of *A. marina* were reported to have identical diagnostic sequences in the V1 region of their 16S rRNA genes; the V1 region is longer than those of any other cyanobacteria (12, 16). Hence, we designed a primer, namely, AcaryoSPF, specific to *Acaryochloris* spp., on the basis of the 16S rRNA sequence in the V1 region. CYA781R(b) was selected as the reverse primer to be used in combination with AcaryoSPF, because the former has higher specificity than CYA781R(a). On the basis of the 16S rRNA gene sequence of *A. marina* MBIC11017, the expected length of the DNA fragments amplified with the primers AcaryoSPF and CYA781R(b) was 689 bp.

**Fig. 3:**
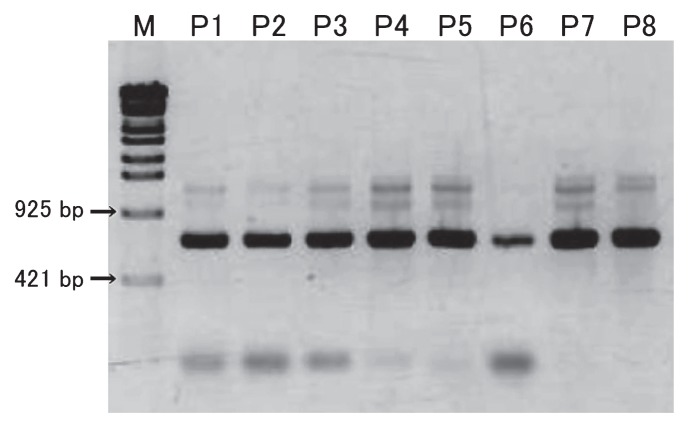
Agarose gel electrophoresis of PCR products with a specific primer set (AcaryoSPF-CYA781R(b)). P1–P7 are samples of didemnid ascidians, and P8 is a sponge sample (Table 1). M: DNA marker (λ StyI)

**Fig. 4:**
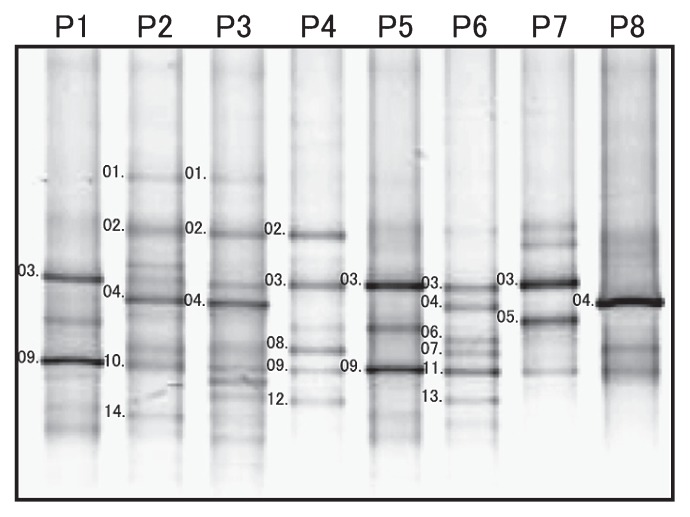
Diversity of *Acaryochloris* phylotypes in didemnid ascidians and a sponge from Palau, which was detected with a specific primer set (AcaryoSPF-CYA781R(b)). P1–P7 are samples of didemnid ascidians, and P8 is a sponge sample (Table 1). Band numbers 01–14 in this figure correspond to bands sp01–sp14, respectively, as described in the text, tables, and figures.

A reaction performed with the primer set AcaryoSPF-CYA781R(b) resulted in the amplification of approximately 0.7-kb DNA fragments from all the invertebrate samples (Fig. 3); this result indicates that the samples contained genetic material of *Acaryochloris* spp. The DNA amplicons obtained in the abovementioned reaction were purified and used as templates for PCR with the BAC341F-CYA781R(b) primer set in order to attach guanine-cytosine (GC) clamps and obtain a short fragment that would be suitable for separation by DGGE. On performing DGGE, 1–6 bands were obtained from each sample, and 14 different sequences were derived from these bands (bands sp01–14 in Fig. 4). The sequence of band sp03 was identical to the 16S rRNA gene of *A. marina* MBIC11017, and all the other bands were remarkably similar to the gene (>98% homology). No other phylotype of cyanobacteria was detected. Further, the bands of *Acaryochloris* spp., except for bands B03 and B10, were newly observed using the primer set AcaryocSPF-CYA781R(b). From these results it was evident that *Acaryochloris* spp. are associated with all the studied invertebrates, and that the detection of *Acaryochloris* spp. was obscured in our previously developed method because of the presence of predominant cyanobacterial symbionts.

### Distribution of *Acaryochloris* phylotypes

One to six bands were obtained from each sample, and band patterns varied (Fig. 4). Although bands sp03 and sp09 were detected in many samples, no band was detected in all samples. Although invertebrate samples P1, P2, and P6 were identified as belonging to *L. patella*, the band patterns derived from these samples were different from one another. In contrast, P2 (*L. patella*) and P3 (*L. punctatum*), which were obtained from different species but collected from the same location, showed very similar band patterns. Additionally, P1 (*L. patella*) and P5 (*Didemnum* sp.), which differed in terms of the genus level, collection site, and predominant cyanobacterial symbionts (*Prochloron* sp. and *Synechocystis trididemni*, respectively), showed identical band patterns (Fig. 4).

### Phylogenetic relationships among *Acaryochloris* spp. in association with invertebrates

**Fig. 5:**
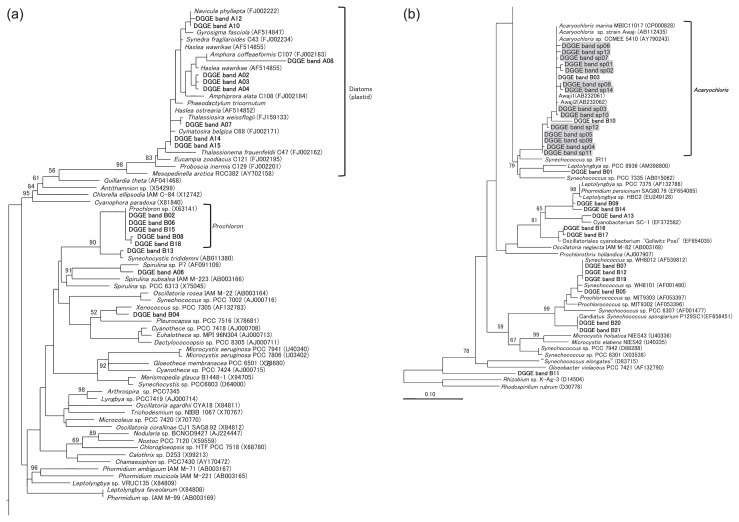
Cyanobacterial phylogenetic tree containing the sequences of the DGGE bands obtained in this study. Bold text indicates DGGE bands obtained with the primer sets BAC341F-CYA781R(a) and BAC341F-CYA781R (b) (Fig. 2), and shaded text indicates DGGE bands obtained with the primer set AcaryoSPF-CYA781R(b) (Fig. 4). Numbers at each node show the neighbor-joining bootstrap values represented as a percentage; nodes without any values received less than 50% support.

**Fig. 6:**
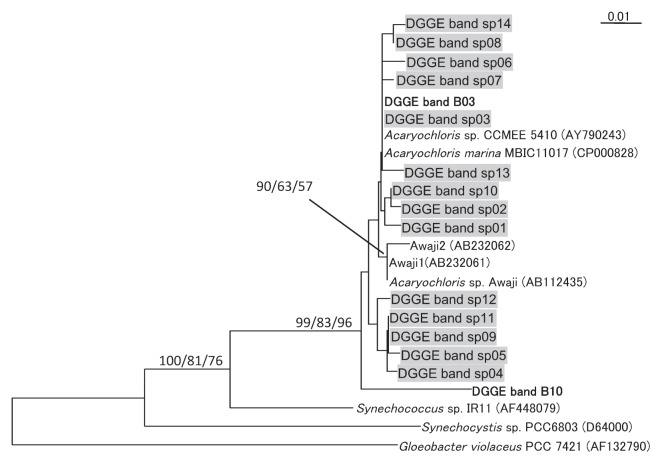
Phylogenetic tree of *Acaryochloris* spp. and their relatives. Bold text indicates DGGE bands obtained with the primer set BAC341F-CYA781R (b) (Fig. 2), and shaded text indicates DGGE bands obtained with the primer set AcaryoSPF-CYA781R(b) (Fig. 4). Numbers at each node (x/y/z) show the neighbor-joining bootstrap values (x), maximum parsimony bootstrap values (y), and maximum likelihood bootstrap values (z). Nodes without any values received less than 50% support in all 3 methods.

The phylogenetic tree shown in Fig. 5 was constructed with the alignment data set derived from the sequences of the DGGE bands, which were classified as belonging to cyanobacteria or algal plastids. Consistent with the results of the BLAST search shown in Table 2, all the sequences of bands sp01–14 (Fig. 4) as well as bands B03 and B10 (Fig. 2) formed an *Acaryochloris* cluster, which independently diverged from other cyanobacteria. Detailed phylogenetic relationships among all the phylotypes in the *Acaryochloris* cluster were also elucidated (Fig. 6). All *Acaryochloris* phylotypes were clustered with a high bootstrap value. Within the cluster, band B10 markedly diverged, while the remaining phylotypes were divided into 2 subgroups: The phylotypes of the 3 clonal strains and the 2 phylotypes obtained from macroalgae collected from Awaji Island (Awaji 1 and 2) belonged to 1 subgroup. The phylotypes obtained from Awaji Island formed a clade; however, the bootstrap values did not support the branching order except in the case of the Awaji group.

## Discussion

### Primer specificity for *Acaryochloris* spp

The *Acaryochloris*-specific primer designed in this study was derived from the 20-nt sequence characteristically found in 16S rRNA genes of *Acaryochloris* spp. but not in any other cyanobacteria (12, 16). This primer enabled selective detection of the *Acaryochloris* phylotypes even in the presence of other predominant cyanobacteria; thus, this primer is effective for the detection of *Acaryochloris* spp. from environmental samples.

However, the relationship between the region of the 16S rRNA gene to which the AcaryoSPF primer binds and the presence of Chl *d* has not been elucidated. The AcaryoSPF sequence is also present in some β-proteobacteria, and it is argued that *Acaryochloris* spp. acquired the sequence by lateral gene transfer (LGT) (12). On the other hand, the Chl *d* biosynthesis mechanism is unclear, but its acquisition might occur independently of that LGT, because it is quite unlikely that the change of 16S rRNA directly affected the chlorophyll metabolism. The problem is that it is unclear which event occurred first. If LGT in the 16S rRNA gene occurred before acquisition of the Chl *d* biosynthesis mechanism, the primer set AcaryoSPF-CYA781R(b) would detect all Chl *d*-containing cyanobacteria, although some of the detected phylotypes would not contain Chl *d*. In contrast, if the acquisition of the Chl *d* biosynthesis mechanism occurred before LGT in the 16S rRNA gene, only some Chl *d*-containing cyanobacteria would be detected with this primer set. Therefore, with the help of molecular analysis, it is necessary to establish a diverse population of clonal cells of Chl *d*-containing cyanobacteria and related strains.

Band B10 (Fig. 2) obtained by performing PCR-DGGE with the primer set BAC341F-CYA781R(b), which was detected in the sample of *D. molle* (P4), showed some specific characteristics. The sequence of the band had 96.8% homology with that of *A. marina* MBIC11017; however, band B10 was not detected by the specific primer set (Fig. 4). This phylotype was thought to be different from *A. marina* at the species level, since the sequence homology was less than 97.5% (25). Phylogenetic analysis performed using the neighbor-joining method indicated that band B10 represents a sister group of the *Acaryochloris* cluster, although the branching order was not supported by the results of bootstrap analyses (Fig. 6). These findings indicate 2 possibilities: 1. LGT occurred after diversification of this phylotype, and 2. the region of the 16S rRNA gene to which AcaryoSPF binds has been modified because of its high variability. The establishment of clonal cells of this phylotype might help to clarify this specific trait as well as the evolutionary process of the *Acaryochloris* genus. It would appear that the specificity of AcaryoSPF was limited to one or a few species of the genus *Acaryochloris*.

### Cyanobacterial communities in colonial ascidians and sponge

This is the first report describing microalgal and cyanobacterial communities associated with didemnid ascidians and sponges, and the genetic diversity of *Acaryochloris* spp. that exist in association with the above-mentioned invertebrates. Our previously developed method using 3 primer sets, BAC341F-CYA781R, BAC341F-CYA781R(a), and BA341F-CYA781R(b) (22), was used in this study. Since CYA781R is an equimolar mixed primer of CYA781R(a) and CYA781R(b), the bands in BC lanes were thought to be derived from predominant cyanobacteria and algae in samples. With the exception of samples P1 and P4, ascidians possess a single phylotype of a *Prochloron* sp. or *Synechocystis trididemni* as their predominant symbionts (Fig. 2); this finding is consistent with that of previous reports (18, 24). The predominant phylotype detected in the sponge sample (bands B21 in Fig. 2) was identical to a *Synechococcus* sp., which has been detected in the same sponge species, namely, *Neopetrosia exigua* (formerly *Xestospongia exigua*) (6, 26); these findings suggest that obligate symbiosis had been established between cyanobacteria and their invertebrate hosts.

To investigate the diversity of cyanobacteria and microalgae, it would be useful to use cyanobacteria-specific primers CYA781R(a) and CYA781R(b) individually. Although we could not detect *Acaryochloris* spp. masked by predominant cyanobacteria, other minor organisms could be detected. When there were certain symbiotic cyanobacteria, as in this study, or when chloroplasts of macroalgae greatly affected the results, as in a previous study (22), individual use of those primers will enable the detection of minor organisms. On the other hand, the use of CYA781R can reveal the composition of dominant organisms; therefore, the use of these primers should be selected according to the aims.

This method also efficiently detected minor cyanobacteria and microalgae in ascidians and a sponge. For example, the bands of diatom chloroplasts and *Synechococcus* spp. were detected in BCa and BCb lanes, respectively (Fig. 2). These organisms are known to be the major photosynthetic planktons in seawater. Bands A06 and A13 were related to cyanobacterium BBT and SC-1, respectively, which are filamentous cyanobacteria discovered from the black band disease of corals. Some other filamentous cyanobacteria (bands B01, 14, 16, 17) were also detected from ascidians. There were no specific relationship between these cyanobacteria or diatoms and ascidians; therefore, they could have been detected in these invertebrates incidentally.

Two unclear results were obtained by this study. *Prochloron* spp. were not detected from two ascidians (P1 and P4) as the major bands, although *Prochloron* cells are the predominant symbionts of these ascidians (4, 10). Since the whole ascidian colony was used for DNA extraction, the absence of the DNA of *Prochloron* spp. in the PCR template was unlikely. Despite this, bands originating from the *Leptolyngbya* sp. and a particular *Acaryochloris* sp. were mainly observed in the BC lane (band B09 and B10, respectively). A possible explanation for these results is that the 16S rRNA sequence of the *Prochloron* cells in these ascidians underwent modification, particularly in the primer-binding regions. In fact, it was reported that two 16S rRNA sequences of the *Prochloron* spp. obtained from *D. molle* extracts had lower homology with other phylotypes that are associated with different ascidians (5). The other result was that non-cyanobacterial bacteria were detected in this study. A similar phenomenon occurred in our previous study in the detection of Acaryochloris spp. on macroalgae (22). This result may also be explained by the modification in the primer-binding regions of 16S rRNA sequences in these unclassified bacteria. Although further investigations are required to address these complex phenomena, the specificity of the primer should be constantly checked and improved on the basis of the latest sequence databases.

### Non-cyanobacterial bacteria detected from colonial ascidians and sponge

The results of this study revealed the presence of some unknown bacteria that exist in association with didemnid ascidians or sponges. Although primer set BAC341F-CYA781R was designed specifically for the detection of cyanobacteria (21), some non-cyanobacterial bands were also detected (Fig. 2). In the BCa lanes, bands A01, A05, A09, A11, A16, and A17 were not related to any known sequences from the databases. The most similar sequences showed 85–87% homology, and these phylotypes were not classified into any phylogenetic group (Table 2). The presence of bands A01, A05, and A11, which were identical and were detected from samples of the same host species (*L. patella*), indicated that some specific relationship exists between these bacteria and their ascidian hosts. Although BLAST analysis showed that these band sequences were related to the sequence of an uncultured bacterium clone cDNA G03 with 86.4% homology and the highest E-value (Table 2), when analyzing the first 159 bp of these bands, they were found to show the highest similarity to an uncultured bacterial clone 3-2 (DQ494528) with 96.8% homology. Similarly, bands A09 and A17 showed relatively high sequence similarity to clone 3-2 when the same regions of these sequences were analyzed (91.3 and 88.3%, respectively). The sequence of this uncultured bacterial clone 3-2 was obtained from a Caribbean colonial ascidian, *Ecteinascidia turbinata*, by performing PCR-DGGE (23). These results showed that ascidians host symbiotic bacteria through specific interactions. The sequence of band A18 derived from sponge sample P8 is almost identical to the sequence of an uncultured bacterial clone XB1E09F, which has been reported to be a symbiont of sponge, *Xestospongia muta*(17). These results indicated that some unique bacteria, which possess 16S rRNA sequences that have an affinity for primer set BAC341F-CYA781R, are also associated with didemnid ascidians or sponge.

### Phylogenetic diversity and distribution of *Acaryochloris* spp. associated with colonial ascidians and sponge

In contrast to *Prochloron* spp. or *Synechocystis trididemni*, which were present in monoculture in their hosts, multiple phylotypes of *Acaryochloris* spp. were present in didemnid ascidians. One to six DGGE bands were detected in each didemnid ascidian or sponge sample with the *Acaryochloris*-specific primer set. Moreover, 14 phylotypes of *Acaryochloris* spp. were detected (Fig. 4); thus, they showed more diversity than expected, since all the clonal strains of didemnid ascidians were only classified into 1 phylotype, indicating that most of the detected phylotypes were possibly non-culturable. In those detected phylotypes, however, there may be some intermediate organisms between *Acaryochloris marina* and other cyanobacteria.

No specific relationship was identified between *Acaryochloris* spp. and the genera or species of the colonial ascidians. Phylotypes of *Acaryochloris* spp. were found to be widely distributed regardless of the host species or sample localities. This case is similar to that of the distribution of epiphytic *Acaryochloris* spp. on macroalgae (22), wherein 2 phylotypes of the *Acaryochloris* spp. were distributed in 6 independent species, including red, green, and brown macroalgae. Therefore, these results suggested that *Acaryochloris* spp. did not have a specific relationship with host ascidians and sponges; the invertebrates used in this study are examples of the many hosts that harbor these cyanobacteria. To confirm this, further investigations into the distribution of *Acaryochloris* spp. in other host organisms or other regions are required.

In this study, we developed a primer set specific for *Acaryochloris marina* and its relatives. By performing PCR-DGGE with this primer set, we were able to selectively detect organisms belonging to *Acaryochloris* spp. from samples that contained many other cyanobacterial cells, such as *Prochloron* spp. and *Synechocystis trididemni*. A minimum of 14 phylotypes were detected, which were widely distributed and existed in association with didemnid ascidians and a sponge. All could be classified in the *Acaryochloris* genus into 1 or 2 potential species by molecular phylogenetic analysis. It is likely that these marine invertebrates provide a favorable habitat for *Acaryochloris* spp., without any specific symbiotic relationship. The method developed in this study may be useful in studies pertaining to the diversity and distribution of *Acaryochloris* cells in the environment.

## Figures and Tables

**Table 1:** Samples investigated in this study

Sample	Host species	Location
P1	*Lissoclinum patella*	Milky Way (7°15.96′ N, 134°25.61′ E)
P2	*Lissoclinum patella*	
P3	*Lissoclinum punctatum*	

P4	*Didemnum molle*	Johny’s Dropoff (7°04.57′ N, 134°15.20′ E)

P5	*Didemnum* sp.	Carp island (7°05.53′ N, 134°16.64′ E)
P6	*Lissoclinum patella*	
P7	*Lissoclinum timorense*	

P8	*Neopetrosia exigua* (sponge)	Nikko bay (7°20.22′ N, 134°29.10′ E)

**Table 2:** Sequence similarities of the excised DGGE bands

Band	Most similar sequence (accession No.)	%similarity	Phylogenetic group
A01	uncultured bacterium clone cDNA_G03 (HQ189729)	376/435 (86%)	unclassified
A02	*Amphiprora paludosa* C52 chloroplast (FJ002240)	395/399 (99%)	*Bacillariophyceae*
A03	*Cymbella laevis* strain NJCL21 chloroplast (JF277083)	397/399 (99%)	*Bacillariophyceae*
A04	*Cymbella laevis* strain NJCL21 chloroplast (JF277083)	356/359 (99%)	*Bacillariophyceae*
A05	uncultured bacterium clone cDNA_G03 (HQ189729)	331/387 (86%)	unclassified
A06	cyanobacterium BBT (AY515014)	392/400 (98%)	*Cyanobacteria*
A07	*Thalassiosira oceanica* CCMP1005 chloroplast (GU323224)	367/370 (99%)	*Bacillariophyceae*
A08	*Amphora coffeaeformis* isolate C107 chloroplast (FJ002183)	336/336 (100%)	*Bacillariophyceae*
A09	uncultured bacterium clone cDNA_G03 (HQ189729)	372/437 (85%)	unclassified
A10	*Navicula phyllepta* isolate C15 chloroplast (FJ002222)	294/296 (99%)	*Bacillariophyceae*
A11	uncultured bacterium clone cDNA_G03 (HQ189729)	376/435 (86%)	unclassified
A12	*Cymbella acutea* strain NJCA34 chloroplast (JF277096)	400/402 (99%)	*Bacillariophyceae*
A13	cyanobacterium SC-1 (EF372582)	394/401 (98%)	*Cyanobacteria*
A14	*Synedra fragilaroides* isolate C43 chloroplast (FJ002234)	399/402 (99%)	*Bacillariophyceae*
A15	*Synedra fragilaroides* isolate C43 chloroplast (FJ002234)	397/399 (99%)	*Bacillariophyceae*
A16	uncultured bacterium clone 300D3.5 (JF827525)	375/429 (87%)	unclassified
A17	uncultured spirochete SRODG048 (FM995181)	368/432 (85%)	Spirochaetes
A18	uncultured bacterium clone XB1E09F (HQ270287)	421/424 (99%)	unclassified
B01	*Leptolyngbya* sp. PCC 8936 (AM398800)	371/400 (92%)	*Cyanobacteria*
B02	*Prochloron* sp. (X63141)	400/400 (100%)	*Cyanobacteria*
B03	*Acaryochloris marina* MBIC11017 (CP000828)	369/369 (100%)	*Cyanobacteria*
B04	*Xenococcus* sp. PCC 7305 (AF132783)	391/400 (97%)	*Cyanobacteria*
B05	*Synechococcus* sp. WH 8012 (AF539812)	392/400 (98%)	*Cyanobacteria*
B06	*Prochloron* sp. (X63141)	392/392 (100%)	*Cyanobacteria*
B07	*Synechococcus* sp. WH 8012 (AF539812)	400/400 (100%)	*Cyanobacteria*
B08	*Prochloron* sp. (X63141)	398/400 (99%)	*Cyanobacteria*
B09	*Leptolyngbya* sp. HBC2 (EU249128)	396/400 (99%)	*Cyanobacteria*
B10	*Acaryochloris marina* MBIC11017 (CP000828)	387/400 (96%)	*Cyanobacteria*
B11	uncultured bacterium clone F66 (JN379054)	371/399 (93%)	unclassified
B12	*Synechococcus* sp. KUAC 3041 (EF152371)	389/389 (100%)	*Cyanobacteria*
B13	*Synechocystis trididemni* (AB011380)	367/376 (97%)	*Cyanobacteria*
B14	*Phormidium persicinum* SAG 80.79 (EF654085)	390/400 (97%)	*Cyanobacteria*
B15	*Prochloron* sp. (X63141)	382/384 (99%)	*Cyanobacteria*
B16	Oscillatoriales cyanobacterium ‘Gollwitz Poel’ (EF654035)	396/400 (99%)	*Cyanobacteria*
B17	Oscillatoriales cyanobacterium ‘Gollwitz Poel’ (EF654035)	392/400 (98%)	*Cyanobacteria*
B18	*Prochloron* sp. (X63141)	362/363 (99%)	*Cyanobacteria*
B19	*Synechococcus* sp. KUAC 3041 (EF152371)	365/365 (100%)	*Cyanobacteria*
B20	*candidatus* Synechococcus spongiarum P129SC1 (EF656451)	399/400 (99%)	*Cyanobacteria*
B21	*candidatus* Synechococcus spongiarum P129SC1 (EF656451)	400/400 (100%)	*Cyanobacteria*
sp01	*Acaryochloris marina* MBIC11017 (CP000828)	390/392 (99%)	*Cyanobacteria*
sp02	*Acaryochloris marina* MBIC11017 (CP000828)	390/392 (99%)	*Cyanobacteria*
sp03	*Acaryochloris marina* MBIC11017 (CP000828)	392/392 (100%)	*Cyanobacteria*
sp04	*Acaryochloris marina* MBIC11017 (CP000828)	388/392 (98%)	*Cyanobacteria*
sp05	*Acaryochloris marina* MBIC11017 (CP000828)	387/392 (98%)	*Cyanobacteria*
sp06	*Acaryochloris marina* MBIC11017 (CP000828)	390/392 (99%)	*Cyanobacteria*
sp07	*Acaryochloris marina* MBIC11017 (CP000828)	391/392 (99%)	*Cyanobacteria*
sp08	*Acaryochloris marina* MBIC11017 (CP000828)	390/392 (99%)	*Cyanobacteria*
sp09	*Acaryochloris marina* MBIC11017 (CP000828)	388/392 (98%)	*Cyanobacteria*
sp10	*Acaryochloris marina* MBIC11017 (CP000828)	391/392 (99%)	*Cyanobacteria*
sp11	*Acaryochloris marina* MBIC11017 (CP000828)	389/392 (99%)	*Cyanobacteria*
sp12	*Acaryochloris marina* MBIC11017 (CP000828)	388/392 (98%)	*Cyanobacteria*
sp13	*Acaryochloris marina* MBIC11017 (CP000828)	390/392 (99%)	*Cyanobacteria*
sp14	*Acaryochloris marina* MBIC11017 (CP000828)	390/392 (99%)	*Cyanobacteria*
